# Circulating MicroRNAs: Potential Biomarkers for Cancer

**DOI:** 10.3390/ijms12032055

**Published:** 2011-03-22

**Authors:** De-Cai Yu, Qing-Guo Li, Xi-Wei Ding, Yi-Tao Ding

**Affiliations:** 1 Institute of Hepatobiliary Surgery, Nanjing University, Nanjing, Jiangsu, China; E-Mails: dryudecai@hotmail.com (D.-C.Y.); lqg0235062@163.com (Q.-G.L.); dxw0008@sina.com (X.-W.D.); 2 Department of Hepatobiliary Surgery, the Affiliated Drum Tower Hospital, School of Medicine, Nanjing University, Nanjing, Jiangsu, China

**Keywords:** circulation, microrna, neoplasm, biomarkers

## Abstract

Cancer is the leading cause of death in the world. Development of minimally invasive biomarkers for early detection of cancer is urgently needed to reduce high morbidity and mortality associated with malignancy. MicroRNAs (miRNAs) are small regulatory RNAs that modulate the activity of specific mRNA targets and play important roles in a wide range of physiologic and pathologic processes. Recently, miRNAs were found to be dysregulated in a variety of diseases including cancer. Emerging evidence suggests that miRNAs are involved in tumor initiation and progression. Together, the different expression profiles of miRNAs in cancer, and the stability of circulating miRNAs, make them new potentially clinical biomarkers for cancer diagnosis, classification, therapeutic decisions, and prognosis.

## 1. Introduction

Cancers are often diagnosed at a late stage with concomitant poor prognosis. The development of minimally invasive tests for the detection and monitoring of common epithelial malignancies could greatly reduce the worldwide health burden of cancer [1]. As the blood is easily accessible and taking a sample is lowly invasive, testable biomarkers found in blood would be especially useful. Conventional strategies for blood-based biomarker discovery (e.g., circulating proteins, DNA, mRNA) have shown promise, but their clinical use has been limited due to lack of sufficient sensitivity, specificity, and stability [1]. New approaches that can complement and improve on current strategies for cancer detection are urgently needed.

MiRNAs are small (typically ∼22 nt in size) regulatory RNA molecules that modulate the activity of specific mRNA targets and play important roles in a wide range of physiologic and pathologic processes [2]. Altered miRNA expression has been reported in various cancers, and the expression patterns of miRNAs in human cancer appear to be tissue-specific [3]. MiRNAs have shown great potential as tissue-based markers for cancer definition. Recently, the findings that human blood contains stably expressed miRNAs have revealed a great potential of circulating miRNA signature as disease fingerprints to predict survival [4]. Due to their size, abundance, tissue specificity, and relative stability in circulation, miRNAs hold promise as unique accessible biomarkers to detect and monitor cancer.

## 2. MiRNA Function

MiRNAs are small (∼22 nt), endogenous, single-stranded, non-coding regulatory RNA molecules. MiRNAs genes often cluster in the genome and are transcribed by RNA polymerase II to form pri-miRNAs in the nucleus. Pri-miRNAs then undergo processing by two cellular nucleases, Drosha and Dicer, to generate mature miRNAs. In animals, miRNAs mostly bind to complementary sequences in the 3′ untranslated region (UTR) of their target mRNAs and negatively regulate gene expression either by translational repression or degradation of the mRNA transcript. MiRBase (Release 14.0), the central database for miRNAs, lists 15,172 entries with 1048 human miRNAs until now [5]. Many miRNAs are well conserved across all species, suggesting a crucial role. Studies have shown that miRNAs are involved in various biologic processes and pathways such as cell proliferation, differentiation, apoptosis, metabolism, and tissue morphogenesis. Recent data suggest a direct link between miRNAs and disease, and miRNA expression signatures are associated with various types of cancer. In addition, the discovery that miRNAs are often expressed in a very tissue-specific manner during development is also suggestive of a crucial role of miRNAs in specifying and maintaining tissue identity.

### 3. Specific Expression of MiRNAs in Cancer

It is now recognized that miRNAs are frequently dysregulated in various cancers [6]. A number of miRNAs were shown to be located in fragile regions of the human genome that are associated with cancer [7]. Functional studies indicate that miRNAs have both tumor suppressive and oncogenic potential in human cancer [8]. Under-expressed miRNAs such as let-7 in lung cancer and miR-15/16 in leukemia are tumor suppressor genes, suppressing Ras and BCL2, respectively [9,10]. Over-expressed miRNAs, such as mir-21 and the cluster mir-17–92, are oncogenes, targeting tumor suppressors PTEN and E2F1 in solid and hematologic malignancies, respectively [11]. Furthermore, dysregulated miRNAs have been shown to play a crucial role in tumor initiation, progression, and metastasis, and are often associated with diagnosis, prognosis, and response to therapy [12–14]. With the development of technologies to detect the expression levels of hundreds of miRNAs at a time and the clear role of miRNAs in cancers, groups began looking at miRNA profiles of different cancers. Lu [15] developed a method for bead-based miRNA profiling. Employing this technique on twenty different cancers, they found that each cancer had a specific miRNA profile and that most poorly differentiated tumors could be classified to their tissues of origin based on their miRNA expression levels. Furthermore, miRNA expression differentiates histology and predicts survival and relapse of lung cancer [9,16,17].

## 4. Circulating MiRNAs: Stable RNA Molecules

Circulating miRNAs are not only abundant in blood, but also very stable, which are important prerequisites as clinical biomarkers. MiRNAs are well resistant to plasma RNases. Mitchell [18] compared the stability of endogenous miRNAs with synthetic miRNAs without homologous sequences into human plasma. The levels of endogenous miRNAs were not significantly altered, while synthetic miRNAs rapidly degraded when added directly to plasma. Chen [19] treated miRNAs with RNase A digestion, while 18s rRNA, 28s rRNA, GAPDH, β-actin, and U6 were used as controls. Surprisingly, more than half of the miRNAs remained intact after 3 h of exposure to RNase A. In contrast, all controls were rapidly degraded by RNase A. Furthermore, miRNAs remain stable after being subjected to harsh conditions including boiling, low/high pH, extended storage, freeze-thaw cycles and so on. MiRNAs also have unusually high stability in formalin-fixed tissues [20]. The mechanism of resistance of miRNAs to RNase requires further studies. Of note, measurements obtained from plasma or serum were strongly correlated, indicating that both serum and plasma samples will be suitable for investigations of miRNAs as blood-based biomarkers [18].

In sum, miRNAs in the circulation are relatively stable, very accessible, low invasive, and easily testable biomarkers.

## 5. The Origin and Function of Circulating MiRNAs in Tumors

Circulating miRNAs in the blood of cancer patients could play the same important roles as miRNAs in tissues. However, the secretory mechanism and biological function of extracellular miRNAs remain unclear. Mitchell [18] used a mouse xenograft model where a human prostate cancer cell line was implanted into mice to show that there were tumor-derived miRNAs circulating in blood. The plasma miR-184 levels were 10-fold reduced after the surgical removal of tumor, which further suggested that miRNAs originated from the primary tumor [21]. Furthermore, employing Solexa deep sequencing, Chen identified specific expression patterns of serum miRNAs for lung cancer and colorectal cancer, providing evidence that both of these two cancers had specific serum-miRNA profiles. These reports clearly show that cancers affect miRNA levels in the circulation, although it is still unclear how tumor miRNAs come into the circulation.

Tumor miRNAs may be present as a result of tumor cells dying and getting lysed or tumor cells releasing exosomes that contain miRNAs. Tumor-derived exosomes are small membrane vesicles of endocytic origin released by the tumor, which may play important roles in cell-cell communication [22]. Ceramide, whose biosynthesis is regulated by neutral sphingomyelinase 2 (nSMase2), triggers secretion of small membrane vesicles called exosomes [23]. Furthermore, Rabinowits [24] demonstrated that there was a close correlation between circulating miRNAs of tumor-derived exosomes and tumor miRNAs in lung adenocarcinoma. Thus, exosomes could be an important source of miRNA in the circulation.

## 6. Circulating MiRNAs: the Biomarkers of Tumors

Through various techniques, numerous groups have shown that different cancer types have distinct miRNA profiles, summarized in Table 1. These findings suggest that circulating miRNAs have potential use as novel biomarkers for diagnosis and prognosis, and may prove useful information in clinical management during the peri-operative period.

Mitchell *et al.* [18] investigated that miRNAs originating from human prostate cancer xenografts enter into the circulation, are readily measured in plasma, and can robustly distinguish xenografted mice from controls. Their concepts extended to cancer in humans, where circulating levels of miR-141 (a miRNA expressed in prostate cancer) can distinguish patients with prostate cancer from healthy controls. Furthermore, the elevated serum levels of miR-200a and miR-200b in most patients with pancreatic cancer could have diagnostic utility [25]. The plasma concentrations of miRNAs (miR-17-5p, miR-21, miR-106a, miR-106b) in gastric cancer [2,26], and miR-184 in squamous cell carcinoma patients [18], and miR-195 and let-7a in breast cancer [27], were significantly higher than controls, and significantly reduced further after the surgical removal of tumor. Otherwise, the relative amount of miR-92a in the plasma from hepatocellular carcinoma (HCC) patients was decreased compared with that from healthy donors, but was elevated after surgical treatment [28] .The reduction or elevation of plasma miRNA levels after the surgical removal of tumor further confirmed the correlation between miRNA and the primary tumors. Furthermore, Heneghan [27] found that specific circulating miRNAs correlated with certain pathological variables, namely nodal status and estrogen receptor status.

Calin *et al.* [29] were the first to show that their miRNA microarray could differentiate between B cell chronic lymphocyte leukemia (CLL) cells and normal cells. Furthermore, they classified CLL samples into two different groups based on their miRNA profiles, and these profiles corresponded to high or low levels of a protein that is associated with a positive prognosis at low levels. The patients with diffuse large B cell lymphoma (DLBCL) had high serum levels of miR-21, which was associated with improved relapse-free survival [30]. This result is consistent with their previous findings in biopsy material from a different cohort of DLBCL patients, where high tumor miR-21 expression was also associated with a more favorable clinical outcome. Most recently, Leidinger [31] screened and filtered 51 differentially regulated miRNAs in blood cells of melanoma patients. With a subset of 16 significantly deregulated miRNAs, the classification accuracy, specificity, and sensitivity reached 97.4%, 95%, and 98.9% by supervised analysis. Hu [32] also found that the miRNA signature from the serum may predict overall survival of non-small cell lung cancer (NSCLC). Eleven serum miRNAs were found to be altered more than five-fold by Solexa sequencing between longer-survival and shorter-survival groups, and levels of four miRNAs (*i.e.*, miR-486, miR-30d, miR-1 and miR-499) were significantly associated with overall survival.

## 7. Perspectives

Together, these results indicate that circulating miRNAs have many characteristics of ideal biomarkers, most notably their inherent stability and resilience. Firstly, miRNA expression is frequently dysregulated in cancer [3,6]. Secondly, expression patterns of miRNAs in human cancer appear to be tissue-specific [15]. Thirdly, miRNAs have unusually high stability [4]. The expression of specific circulating miRNAs is a good surrogate of tumor miRNA expression and initiates a new paradigm that will be useful not only for early diagnosis but also for prognostic and therapeutic decisions [51].

Furthermore, a lot of challenges regarding miRNAs in sera need to be confronted. Firstly, the specificity of miRNAs: one miRNA can distinguish different cancers which have the same serum miRNA, e.g., miR-21 in DLBCL and pancreatic cancer. Secondly, the standardization of miRNAs: the preparation of serum/plasma will need be standardized in order to generalize findings from different patients, groups, or labs. Thirdly, the quantification of miRNAs: what will be used as a standard for qRT-PCR for measuring circulating miRNAs (e.g., as “housekeeping” serum miRNA/small RNA), as other classes of RNAs or mRNAs are not stable in serum? Apart from these issues, questions about the biological half-life of the circulating miRs need to be addressed, as this has critical effects in clinical applications. Therefore, the functional roles of miRNAs in tumor biology are currently being unraveled and worth further investigations.

## Figures and Tables

**Table 1.** Circulating miRNAs as biomarkers of tumor.

**Tumor Type**	**MicroRNAs**	**Significance**	**References**
Breast cancer	miR-195 and let-7a; miRNA-21, 106a, 126, 155, 199a and 335	Correlated with nodal status and estrogen receptor status, and decreased postoperatively	[27,33]
CRC	miR-92; miR-29a and miR-92a	Diagnosis with 70% specificity and 89% sensitivity; early detection of CRC	[34,35]
DLBCL	miR-21	Relapse-free survival	[30]
Gastric cancer	miR-17-5p, miR-21, miR-106a, miR-106b, let-7a; miR-1, miR-20a, miR-27a, miR-34 and miR-423-5p	Up or down-regulated; Correlated to tumor stage	[26,36]
HCC	miR-92a; miR-500; miR-25, miR-375, and let-7f	Down-regulated, up-regulated after surgical treatment, and diagnosis	[28,37,38]
Leukemia	miR-92a/miR-638; miR-29a, -181a, and -221	Diagnosis and treatment	[39,40]
Melanoma	gene profiles	21 miRNAs down-regulated/30 miRNAs up-regulated	[31]
MM	miR-193b-365	Up-regulated	[41]
NSCLC	miR-25 and miR-223;exosomal miRNA; miR-486, miR-30d, miR-1 and miR-499	Up-regulated; screening test; overall survival	[19,24,32]
Oral cancer	miR-31; miR-184	Up-regulated, and down-regulated after surgical resection.; diagnosis	[21,42]
Ovarian cancer	miRNAs-21, 92, 93, 126 and 29a; MiRNAs-155, 127 and 99b	Up-regulated, and related with pre-operative CA-125; down-regulated; exosomes	[43–45]
Pancreatic cancer	miR-21, miR-210, miR-155, and miR-196a	Diagnosis	[25,46–48]
Prostate cancer	miR-141	Diagnosis	[18]
ESCC	miR-10a, miR-22, miR-100, miR-148b, miR-223, miR-133a, and miR-127-3p	Up-regulated	[49]
Rhabdomyosarcoma	miR-206	Up-regulated	[50]

CRC: colorectal carcinoma; DLBCL: diffuse large B cell lymphoma; HCC: hepatocellular carcinoma; MM: Multiple myeloma; ESCC: esophageal squamous cell carcinoma.
